# Generation and Characterization of *Fmr1* Knockout Zebrafish

**DOI:** 10.1371/journal.pone.0007910

**Published:** 2009-11-19

**Authors:** Marjo J. den Broeder, Herma van der Linde, Judith R. Brouwer, Ben A. Oostra, Rob Willemsen, René F. Ketting

**Affiliations:** 1 Hubrecht Institute, Royal Academy of Arts and Sciences and University Medical Center Utrecht, Utrecht, The Netherlands; 2 Erasmus Medical Center, CBG-Department of Clinical Genetics, Rotterdam, The Netherlands; University of Birmingham, United Kingdom

## Abstract

Fragile X syndrome (FXS) is one of the most common known causes of inherited mental retardation. The gene mutated in FXS is named *FMR1*, and is well conserved from human to *Drosophila*. In order to generate a genetic tool to study *FMR1* function during vertebrate development, we generated two mutant alleles of the *fmr1* gene in zebrafish. Both alleles produce no detectable Fmr protein, and produce viable and fertile progeny with lack of obvious phenotypic features. This is in sharp contrast to published results based on morpholino mediated knock-down of *fmr1*, reporting defects in craniofacial development and neuronal branching in embryos. These phenotypes we specifically addressed in our knock-out animals, revealing no significant deviations from wild-type animals, suggesting that the published morpholino based *fmr1* phenotypes are potential experimental artifacts. Therefore, their relation to *fmr1* biology is questionable and morpholino induced *fmr1* phenotypes should be avoided in screens for potential drugs suitable for the treatment of FXS. Importantly, a true genetic zebrafish model is now available which can be used to study FXS and to derive potential drugs for FXS treatment.

## Introduction

Fragile X syndrome (FXS) is one of the most common known causes of inherited mental retardation with a frequency of 1∶4000 males and 1∶6000 females [1]. In almost all cases, FXS is due to the expansion of the unstable CGG trinucleotide repeat sequence in the 5′ untranslated region of the *FMR1* gene [2], [3]. Once the repeats exceed 200 units (full mutation), the gene is silenced due to the consequent hypermethylation of the CpG island and CGG repeat. Thus, no mRNA is produced, and the lack of the gene product, FMRP, is responsible for the mental retardation in fragile X patients [4]. Other clinical features include macroorchidism, autistic behaviour, epileptic seizures, hyperactivity, attention deficits and mild craniofacial abnormalities [1].

FMRP is a ubiquitously expressed RNA-binding protein, including two KH domains and an RGG box, with high expression levels in brain and testis [5], [6]. The protein can bind to RNAs containing a G-quartet structure and forms together with many other mRNAs and proteins a messenger ribonucleoprotein (mRNP) particle [7], [8]. The dynamics and transport of mRNP particles over long distances within the dendrites of neurons is established by movement along microtubules [9].

The development of mouse models of FXS has facilitated cellular studies on the underlying molecular basis of this loss-of-function disorder [10], [11]. *Fmr1* knock-out mice recapitulate the typical characteristics of FXS, including behavioural abnormalities, learning deficits and audiogenic seizures. Microscopic analysis of brain material from both FXS patients and *Fmr1* knockout mice has shown dendritic spine abnormalities [12]–[17]. The discovery of a spine morphological phenotype indicates a possible defect in synaptic plasticity in FXS. The precise physiological function of FMRP is still not defined; therefore, the role of FMRP at the synapse has become a central research interest. Compelling evidence predicts a model in which FMRP is involved in the regulation (repression) of local protein synthesis at the synapse, which is triggered group 1 mGluR (mGluR1 and mGluR5) activation. Thus, a lack of FMRP may lead to uncontrolled (exaggerated) protein synthesis at the synapse upon group 1 mGluR stimulation and may underlie the enhanced hippocampal and cerebellar LTD found in *Fmr1* knock-out mice [16], [18], [19]. Interestingly, some behavioural abnormalities could be rescued in *Fmr1* knock-out mice using mGluR5 antagonists [20], [21]. Recently, a rescue of the spine morphological phenotype could be established in cultured *Fmr1* knock-out hippocampal neurons using two different mGluR5 antagonists [21].

In 2006, Tucker *et al*. reported the use of zebrafish embryos to model FXS [22]. Instead of a knock-out approach, a knock-down strategy was applied using microinjection of morpholinos (MOs) into 1–2 cell stage embryos. MOs are antisense oligonucleotides, in which the deoxyribose is substituted with an N-morpholino ring. They can bind to a target mRNA and prevent either translation or normal splicing for up to 4 days. Hence, inhibition of translation is transient and may not result in a complete loss-of-function. Injection of *fmr1* specific MOs resulted in abnormal axonal branching, changes in trigeminal ganglion number and craniofacial abnormalities. Most of these abnormalities in zebrafish embryos could be rescued using MPEP, an mGluR5 antagonist, or by *fmr1* overexpression [22].

In the present study, we generated two independent *fmr1* knock-out alleles using TILLING (targeted induced local lesions in genomes). TILLING combines random induced mutations by ENU treatment and subsequent screening for null mutations [23]. We provide a characterization of both homozygous and transheterozygous mutants with special emphasis on the phenotypic features reported earlier in the *fmr1* knock-down study [22].

## Results

### Isolation of Two Fmr1 Mutant Alleles

**Figure 1:**
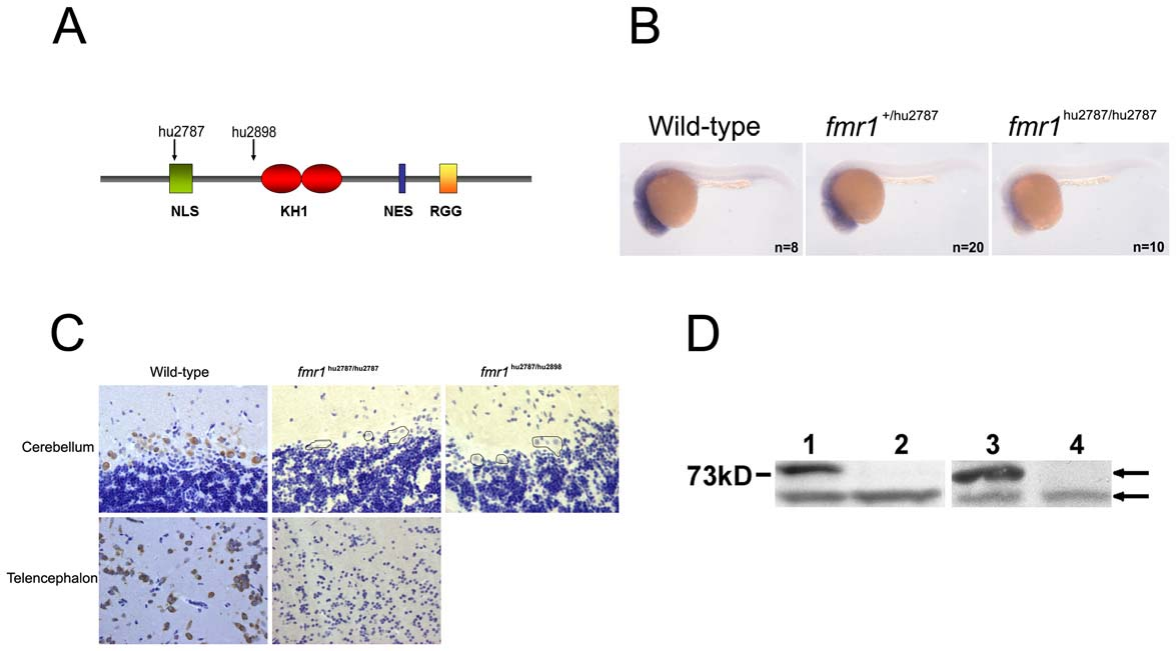
*Fmr1* mutant alleles. A) Illustration of the *fmr1* gene product. The different domains are indicated, along with the sites where isolated mutant alleles will affect the protein. B) Whole mount *in situ* hybridisation with an *fmr1* specific probe. C) Immuno staining of wild type and mutant brain sections using Fmr specific antibodies. Some Purkinje cells in the mutant have been outlined. D) Brain lysates were analyzed by western blot, using an Fmr specific antibody. Lanes 1 and 3 contain wild type samples. Lane 2 contains *hu2787/hu2787* lysate. Lane 4 contains *hu2787/hu2898* lysate. The upper arrow points at Fmr. The lower arrow points at an a-specific band that serves as a loading control.

In order to develop a genetic model in which the effects of FMRP on brain development can be easily studied during development we screened for knock-out alleles in the zebrafish system. From a randomly mutagenized library we isolated two independent mutant alleles: hu2787 defines a C to T change in the coding region of *fmr1* (ENSDARG00000037433), leading to the introduction of a premature stop at codon position 113 (Figure 1A). The mRNA derived from this allele is less stable than that derived from the wild-type *fmr1* locus. This is illustrated in Figure 1B using whole mount *in situ* hybridisation with an *fmr1* specific probe, on a batch of embryos obtained from a cross between heterozygous parents. Presumably this is the result from a well-known phenomenon named nonsense-mediated-decay (NMD). Furthermore, using a C-terminal antibody we are unable to detect expression of Fmr in neurons using immunocytochemistry on paraffin sections, whereas a high expression could be detected in neurons from wild type zebrafish. Figure 1C illustrates high Fmr expression in Purkinje cells in the cerebellum and neurons in the telencephalon. In addition, we determined Fmr expression in total brain homogenates by Western blot analysis. Consistent with the immuno-stainings, no Fmr was detectable (Figure 1D). The second allele we isolated, hu2898, has a mutated splice acceptor site at the end of the 7th intron. Sequencing of splicing products from this allele shows that hu2898 leads to the use of an alternative splice acceptor site 2 bases downstream of the original site. This induces a frameshift with regard to the original reading frame and an opal stop codon 27 nucleotides downstream (Figure 1A). Animals carrying any combination of the two mutant alleles show loss of Fmr in both immunocytochemistry (Figure 1C) and Western blot analysis (Figure 1D).

### Fmr1 Mutant Zebrafish Are Viable

Animals lacking zygotic Fmr are found at Mendelian frequencies in crosses between heterozygous parents. They display wild-type development, and develop into fertile adults with no gross abnormalities. Progeny from homozygous mutant parents were also analyzed to check the potential effect of maternally provided protein and/or mRNA on development. Also these maternal-zygotic (MZ) mutant animals develop normally, and display no obvious defects in behaviour or fertility. Importantly, we did not observe selective pressure against homozygous mutant combinations in any of the crosses we performed (not shown), strongly suggesting that potentially lethal phenotypes are not repressed by the presence of genetic modifiers in our genetic backgrounds.

### Lack of Fmr1 Does Not Induce Craniofacial Defects

**Figure 2:**
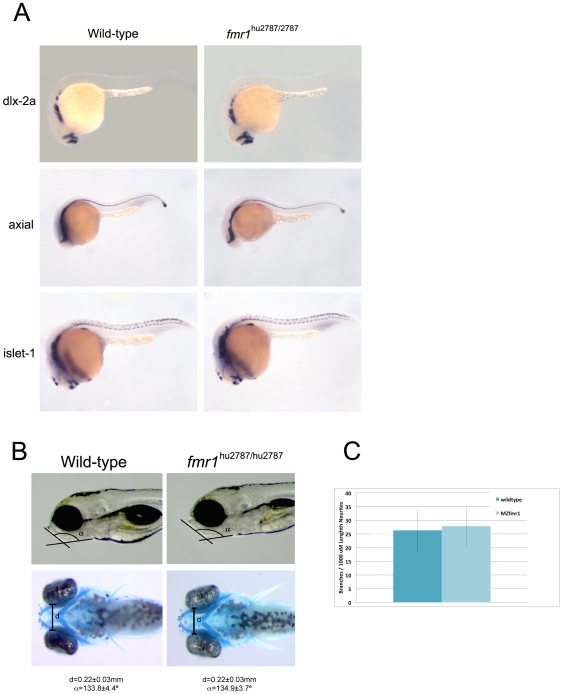
Phenotypic assays on wild-type and *fmr1* mutant embryos. A) Wild type and mutant embryos were analyzed using whole mount *in situ* hybridisation using probes against *dlx-2a*, *axial* and *islet-1*. B) The width of Meckel's cartilage was measured in wild type (n = 9) and MZ *fmr1* mutant (n = 11) embryos. The angle of this structure with regard to the anterior-posterior axis was also measured in wild-type (n = 6) and *fmr1* mutant (n = 9) embryos. Indicated errors represent SD. C) Neurite branching was measured on Rohon-Beard neurites using the monoclonal antibody zn-12. Plotted is the branching frequency per 1000 µm in both wild-type and MZ stop mutant embryos. In total n = 25 neurites (wild-type) and n = 28 neurites (MZfmr1) were traced in a total of 8 embryos of each genotype. Error bars represent SD.

The results described above contrasts with morpholino induced *fmr1* knock-down studies that have been published before [22]. More specifically, it was demonstrated that these morphants display aberrant expression of three markers: *axial*, *dlx-2a* and *islet-1*. We therefore analyzed the expression of these genes by *in situ* hybridisation in *fmr1* MZ null embryos. The results of these experiments are depicted in Figure 2A. We observed no significant differences between wild-type and *fmr1* null embryos in any of the analyses.

In addition, we measured the width of Meckel's cartilage and the angle it makes to the anterior-posterior axis, to address whether these mutant animals develop abnormalities that may be related to the craniofacial defects seen in fragile X patients, as described in the morpholino knockdown morphants [22]. In both the MZ homozygous stop mutants and embryos derived from homozygous stop mutant mothers and homozygous splice mutant fathers (not shown) the width as well as the angle of this structure is indistinguishable from that in wild-type animals (Figure 2B).

### Fmr1 Mutants Do Not Show Rohon-Beard Neurite Branching Defects

Finally, Tucker *et al* [22] described a defect in neurite branching in Rohon-Beard neurons in *fmr1* morphant embryo's. We therefore analyzed the branching frequency of the Rohon-Beard neurons, similar to what was reported by Tucker *et al* [22]. In Figure 2C we show that also in this analysis we find no significant difference between wild-type and MZ *fmr1* stop mutant animals.

## Discussion

We describe the generation of two *fmr1* knockout alleles in zebrafish, and as such provide a new genetic model system to study FXS, a highly prevalent form of inherited mental retardation. FXS is caused by the loss of the gene product of *fmr1*, Fmr. FXS models have been described in multiple systems and from these models it has become clear that FMRP is acting at the synapse to regulate the translation of target mRNAs upon group 1 mGluR stimulation and whose protein products mediate synaptic strength [16], [18]. *Fmr1* knock-out mice exhibit exaggerated translation of target mRNAs at the synapse. Potentially, such a process can be well affected by mGluR antagonists that would ameliorate the phenotypic outcome of FXS [24].

Establishing a zebrafish model for FXS is very useful in this context, as the zebrafish embryo is amenable to large scale, small molecule drug screens. Supporting this idea was the finding that morpholino induced knock-down of *fmr1* (*fmr1* morphant) in the zebrafish led to embryonic phenotypes that could in principle be used as a read-out in drug screens [22]. Tucker *et al.* [22] reported neurite branching defects and changes in trigeminal ganglion neuron number following *fmr1* knock down [22]. Interestingly, treatment with MPEP, an mGluR5 antagonist, rescued most of these abnormalities, indicating a connection between mGluR5 signalling and *fmr1* function in neurite branching and number of trigeminal ganglion neurons. Furthermore, it suggested that small molecule drug screens in the zebrafish may indeed be an affective manner of finding bio-active lead-compounds that are good starting points for developing drugs beneficial for FXS patients.

Tucker et al [22] also reported craniofacial dysmorphology as a result of *fmr1* knock-down, and this again could be rescued by treatment with MPEP. This is a curious finding, since the role of synaptic connections between neurons in cranial cartilage development is to date totally unexplored, and hence it is not clear whether indeed MPEP would be expected to affect craniofacial defects caused by loss of Fmr.

We here characterize two *fmr1* mutant alleles in the zebrafish, both of which lead to loss of detectable Fmr. In contrast to the above-mentioned MO study, however, we cannot find any gross phenotypical effects caused by these alleles. We checked specifically the above-mentioned phenotypes, craniofacial abnormalities and neurite branching phenotypes, but find no significant differences between wild type and mutant siblings. *Fmr1* mutant fish are also completely fertile, and incrosses between homozygous mutant males and females result in normally developing embryos, indicating that maternally provided mRNA and/or protein is not rescuing first generation *fmr1* mutants.

What could be causing the observed phenotypes in the morphants [22], when genetic *fmr1* null animals do not display these defects? First we explore why genetic mutation of *fmr1* may miss FXS-relevant phenotypes. Redundancy could potentially be an issue. The morpholinos used could affect the *fmr1* homologues *fxr1* and *fxr2*, which are both present in zebrafish. This seems unlikely, however, given the fact the sequence comparison between the morpholino and *fxr-1/2* genes shows very little complementarity. *Fmr1* itself could be duplicated in zebrafish. However, the most recent genome annotation shows no indication of a duplicated *fmr1* gene, and on western blot we detect no protein in *fmr1* homozygous mutant tissue. This makes the presence of a closely related, functional *fmr1* copy unlikely. Finally, potential phenotypes may be rescued by modifier loci; loci that genetically interact with *fmr1* and of which particular alleles may suppress phenotypes triggered by loss of Fmr. Despite the fact that our zebrafish strains show no sign of selection for or against homozygous *fmr1* mutants, this is an option that is difficult to eliminate. Extensive outcrossing into the zebrafish strains used in the studies by Tucker *et al* [22] would be required to test this hypothesis.

There is, however, a more likely potential explanation: the morpholino-induced phenotypes may not be related to loss of Fmr. Morpholino oligonucleotides are well known to cause phenotypes unrelated to knock-down of the intended gene. In fact, 15–20% of MOs used in zebrafish show off-targeting effects that are mediated by p53-induced apoptosis [25]. In the study from Tucker et al. [22] the number of analyzed morphants is very limited. For instance, altered *dlx-2a*, *fgfr1* and *axial* expression could only be observed in 17/30, 11/30 and 3/30 *fmr1* morphants, respectively; for neurite branching phenotypes no numbers are given related to the penetrance of the defect; injection of antibodies against alpha-acetylated tubulin resulted in a dramatic axon defect only in 3/30 and axon defasciculation in 13/30 *fmr1* morphants. Finally, the craniofacial dysmorphology could only be observed in 9/15 *fmr1* morphants.

In summary, we find the loss of *fmr1* in zebrafish at most induces very subtle phenotypes that are not readily detectable using light-microscopy and techniques like immunocytochemistry and *in situ* hybridisation, at least in the strains used in our laboratory. It remains well possible that subtle defects are induced by lesions in *fmr1*, and that these may be used to develop sensitive and robust essays to probe *fmr1* function, which may in turn be used for screening of small molecules libraries in order to find drugs suitable for treatment of FXS. At present, however, we have to conclude that the phenotypes as described by Tucker *et al* [22] may be based on morpholino induced artefacts, and as such not useful to study *fmr1* function in the zebrafish.

## Materials and Methods

### Zebrafish Strains and Screening F1 ENU- Mutation Library

Adult zebrafish were bred and maintained under standard conditions. Staging of embryos was according to Kimmel *et al*. [26]. Embryos at different developmental stages were fixed with 4% PFA/PBS overnight.

ENU induced mutation library was screened for a mutation in the *fmr1* gene. Amplicons were designed for exon 5–6 and exon 7–9 and screened for mutation as described [23]. Fish with mutant alleles (*fmr1* ^hu2787^ (stop); *fmr1^hu2898^* (splice)) were outcrossed against TL and crossed to obtain homozygous or transheterozygous embryos.

### Immunocytochemistry Adult Brain

Adult zebrafish were sacrificed by euthanasia using high dose of MS222, brains were dissected immediately and fixed overnight in 3% paraformaldehyde. The brains were embedded in paraffin according to standard protocols. Sections (7 µm) were deparaffinized, followed by antigen retrieval using microwave treatment in 0.01 M sodium citrate solution. Endogenous peroxidase activity blocking and immunoincubation was performed as described before using polyclonal rabbit 758 antibodies against zebrafish Fmrp [27].

### Western Blotting

Half brains (saggital) from adult zebrafish were homogenised in 500 µl HEPES-buffer (10 mM HEPES, 300 mM KCl, 3 mM MgCl_2_, 100 µM CaCl_2_, 0.45% Triton X-100 and 0.05% Tween-20, pH 7.6, with Complete protease inhibitor cocktail (Roche Diagnostics), while kept on ice. After incubating the homogenates on ice for 30 minutes, they were sonicated twice for 20 seconds. Cell debris was spun down and the supernatant was collected. Loading mix was added to 100 µg of protein, heated at 95°C for 5 minutes and loaded onto a 10% SDS-PAGE gel. After electroblotting the gel onto a nitrocellulose membrane, the membrane was incubated overnight at 4°C with the rabbit polyclonal 758 antibody specific for zebrafish FMRP [27], in PBS-T with 5% milk powder. The next day the membrane was incubated with a horseradish peroxidase conjugated secondary antibody rabbit-α-mouse (DAKO), allowing chemiluminescence detection with an ECL KIT (Amersham).

### In Situ Hybridisation

ISH experiment were performed as described in Thisse et al. [28]. The RNA-probes were made according standard protocols. Probes against *fmr1* (EST-clone fy56do3.x1; IRBOp991C1010D from RZPD, Berlin, Germany) from which the cDNA fragment was cloned into pCS2plus;dlx-2a [29]; axial [30] and islet-1 [31] were used in the described experiments. ISH to show NMD on the *hu2787 fmr1* allele was done in one batch, so that wild-type, heterozygous and homozygous mutant embryos received identical treatments. Embryos were genotyped afterwards, revealing a consistent loss of *fmr1* mRNA in homozygous *hu2787* mutants.

### Cartilage Staining

*fmr1* ^hu2787^ incross embryos (5 dpf) were Alcian blue stained according to Neuhauss et al. [32]. The width and the angle of Meckel's cartilage were measured and embryos were genotyped.

### Antibody Staining Embryos

For whole-mount immunohistochemistry, embryos were fixed in 4% paraformaldehyde for four hours at RT, washed with PBT and incubated overnight at 65°C in FST solution (50% formamide, 2x SSC, 0.1% Tween-20). Next day, washed in PBT and blocked in ABS (PBT, 2% DMSO, 0.1% IGEPAL, 2% lamb serum, 2% BSA) and incubated overnight with the monoclonal antibody zn-12 (1∶200, Developmental Studies Hybridoma Bank). Primary antibody was washed off by ABS buffer, and embryos were incubated overnight with secondary antibody goat anti-mouse conjugated with Alexa-488 (1∶250, Molecular Probe)[Tucker et al, 2006]. Embryos were imaged with a Leica DM6000 microscope, Leica camera DFC 360 FX and Leica LAS AF Software. Images were analysed using the NeuronJ plugin [33].

## References

[1] Hagerman RJ, Hagerman RJ, Hagerman P  (2002). The physical and behavioural phenotype.. Fragile-X syndrome: diagnosis, treatment and research.

[2] Verkerk AJ, Pieretti M, Sutcliffe JS, Fu YH, Kuhl DP (1991). Identification of a gene (FMR-1) containing a CGG repeat coincident with a breakpoint cluster region exhibiting length variation in fragile X syndrome.. Cell.

[3] Oberlé I, Rousseau F, Heitz D, Kretz C, Devys D (1991). Instability of a 550-base pair DNA segment and abnormal methylation in fragile X syndrome.. Science.

[4] Verheij C, Bakker CE, de Graaff E, Keulemans J, Willemsen R (1993). Characterization and localization of the FMR-1 gene product associated with fragile X syndrome.. Nature.

[5] Devys D, Lutz Y, Rouyer N, Bellocq JP, Mandel JL (1993). The FMR-1 protein is cytoplasmic, most abundant in neurons and appears normal in carriers of a fragile X premutation.. Nat Genet.

[6] Tamanini F, Willemsen R, van Unen L, Bontekoe C, Galjaard H (1997). Differential expression of FMR1, FXR1 and FXR2 proteins in human brain and testis.. Hum Mol Genet.

[7] Schaeffer C, Bardoni B, Mandel JL, Ehresmann B, Ehresmann C (2001). The fragile X mental retardation protein binds specifically to its mRNA via a purine quartet motif.. Embo J.

[8] Darnell JC, Jensen KB, Jin P, Brown V, Warren ST (2001). Fragile X Mental Retardation Protein Targets G Quartet mRNAs Important for Neuronal Function.. Cell.

[9] De Diego Otero Y, Severijnen LA, Van Cappellen G, Schrier M, Oostra B (2002). Transport of Fragile X Mental Retardation Protein via Granules in Neurites of PC12 Cells.. Mol Cell Biol.

[10] Bakker CE, Verheij C, Willemsen R, Vanderhelm R, Oerlemans F (1994). Fmr1 knockout mice: A model to study fragile X mental retardation.. Cell.

[11] Mientjes EJ, Nieuwenhuizen I, Kirkpatrick L, Zu T, Hoogeveen-Westerveld M (2006). The generation of a conditional Fmr1 knock out mouse model to study Fmrp function in vivo.. Neurobiol Dis.

[12] Hinton VJ, Brown WT, Wisniewski K, Rudelli RD (1991). Analysis of neocortex in three males with the fragile X syndrome.. Am J Med Genet.

[13] Comery TA, Harris JB, Willems PJ, Oostra BA, Irwin SA (1997). Abnormal dendritic spines in fragile X knockout mice: Maturation and pruning deficits.. Proc Natl Acad Sci U S A.

[14] Nimchinsky EA, Oberlander AM, Svoboda K (2001). Abnormal development of dendritic spines in fmr1 knock-out mice.. J Neurosci.

[15] Galvez R, Gopal AR, Greenough WT (2003). Somatosensory cortical barrel dendritic abnormalities in a mouse model of the fragile X mental retardation syndrome.. Brain Res.

[16] Koekkoek SK, Yamaguchi K, Milojkovic BA, Dortland BR, Ruigrok TJ (2005). Deletion of FMR1 in Purkinje Cells Enhances Parallel Fiber LTD, Enlarges Spines, and Attenuates Cerebellar Eyelid Conditioning in Fragile X Syndrome.. Neuron.

[17] Grossman AW, Aldridge GM, Weiler IJ, Greenough WT (2006). Local protein synthesis and spine morphogenesis: Fragile X syndrome and beyond.. J Neurosci.

[18] Huber KM, Gallagher SM, Warren ST, Bear MF (2002). Altered synaptic plasticity in a mouse model of fragile X mental retardation.. Proc Natl Acad Sci U S A.

[19] Bear MF, Dolen G, Osterweil E, Nagarajan N (2007). Fragile X: Translation in Action.. Neuropsychopharmacology.

[20] Yan QJ, Rammal M, Tranfaglia M, Bauchwitz RP (2005). Suppression of two major Fragile X Syndrome mouse model phenotypes by the mGluR5 antagonist MPEP.. Neuropharmacology.

[21] De Vrij FMS, Levenga J, Van der Linde HC, Koekkoek SK, De Zeeuw CI (2008). Rescue of behavioral phenotype and neuronal protrusion morphology in FMR1 KO mice.. Neurobiol Dis.

[22] Tucker B, Richards RI, Lardelli M (2006). Contribution of mGluR and Fmr1 Functional Pathways to Neurite Morphogenesis, Craniofacial Development and Fragile X Syndrome.. Hum Mol Genet.

[23] Wienholds E, Plasterk RH (2004). Target-selected gene inactivation in zebrafish.. Methods Cell Biol.

[24] Bear MF (2005). Therapeutic implications of the mGluR theory of fragile X mental retardation.. Genes Brain Behav.

[25] Robu ME, Larson JD, Nasevicius A, Beiraghi S, Brenner C (2007). p53 Activation by Knockdown Technologies.. PLoS Genet.

[26] Kimmel CB, Ballard WW, Kimmel SR, Ullmann B, Schilling TF (1995). Stages of embryonic development of the zebrafish.. Dev Dyn.

[27] van 't Padje S, Engels B, Blonden L, Severijnen LA, Verheijen F (2005). Characterisation of Fmrp in zebrafish: evolutionary dynamics of the fmr1 gene.. Dev Genes Evol.

[28] Thisse C, Thisse B, Schilling TF, Postlethwait JH (1993). Structure of the zebrafish snail1 gene and its expression in wild-type, spadetail and no tail mutant embryos.. Development.

[29] Akimenko MA, Ekker M, Wegner J, Lin W, Westerfield M (1994). Combinatorial expression of three zebrafish genes related to distal-less: part of a homeobox gene code for the head.. J Neurosci.

[30] Strahle U, Blader P, Henrique D, Ingham PW (1993). Axial, a zebrafish gene expressed along the developing body axis, shows altered expression in cyclops mutant embryos.. Genes Dev.

[31] Appel B, Korzh V, Glasgow E, Thor S, Edlund T (1995). Motoneuron fate specification revealed by patterned LIM homeobox gene expression in embryonic zebrafish.. Development.

[32] Neuhauss SC, Solnica-Krezel L, Schier AF, Zwartkruis F, Stemple DL (1996). Mutations affecting craniofacial development in zebrafish.. Development.

[33] Meijering E, Jacob M, Sarria JCF, Steiner P, Hirling H, Unser M (2004). Design and Validation of a Tool for Neurite Tracing and Analysis in Fluorescence Microscopy Images.. Cytometry.

